# Bridging the resources gap: deep learning for fluorescein angiography and optical coherence tomography macular thickness map image translation

**DOI:** 10.1186/s12886-022-02577-7

**Published:** 2022-09-01

**Authors:** Hazem Abdelmotaal, Mohamed Sharaf, Wael Soliman, Ehab Wasfi, Salma M. Kedwany

**Affiliations:** Department of Ophthalmology, Faculty of Medicine, Assiut University, Assiut, 71515 Egypt

**Keywords:** Generative adversarial networks image synthesis, Pix2pix, Color-coded macular thickness maps, Fluorescein angiography, Optical coherence tomography

## Abstract

**Background:**

To assess the ability of the pix2pix generative adversarial network (pix2pix GAN) to synthesize clinically useful optical coherence tomography (OCT) color-coded macular thickness maps based on a modest-sized original fluorescein angiography (FA) dataset and the reverse, to be used as a plausible alternative to either imaging technique in patients with diabetic macular edema (DME).

**Methods:**

Original images of 1,195 eyes of 708 nonconsecutive diabetic patients with or without DME were retrospectively analyzed. OCT macular thickness maps and corresponding FA images were preprocessed for use in training and testing the proposed pix2pix GAN. The best quality synthesized images using the test set were selected based on the Fréchet inception distance score, and their quality was studied subjectively by image readers and objectively by calculating the peak signal-to-noise ratio, structural similarity index, and Hamming distance. We also used original and synthesized images in a trained deep convolutional neural network (DCNN) to plot the difference between synthesized images and their ground-truth analogues and calculate the learned perceptual image patch similarity metric.

**Results:**

The pix2pix GAN-synthesized images showed plausible subjectively and objectively assessed quality, which can provide a clinically useful alternative to either image modality.

**Conclusion:**

Using the pix2pix GAN to synthesize mutually dependent OCT color-coded macular thickness maps or FA images can overcome issues related to machine unavailability or clinical situations that preclude the performance of either imaging technique.

**Trial registration:**

ClinicalTrials.gov Identifier: NCT05105620, November 2021. “Retrospectively registered”.

## Background

One of the most common causes of visual impairment in diabetic patients is diabetic macular edema (DME) [1]. Fluorescein angiography (FA) depicts retinal blood flow over time, revealing the status of retinal perfusion and the presence of leakage from the retinal vasculature. Therefore, it plays a crucial role in the staging of diabetic retinopathy (DR) and evaluation of the retinal vasculature. However, the physical characteristics of fluorescein, which can leak from diseased blood vessels obscuring the fluorescence of underlying tissue, and the invasiveness of the technique makes it not without risks [2]. A popular non-invasive method for diagnosing DR and tracking its laser, medicinal, and surgical treatment is optical coherence tomography (OCT). OCT is inherently risk-free and independent of the physical characteristics of fluorescein, as it does not use a dye [3]. OCT offers a quantitative evaluation of DME and the location of retinal thickness. Geographically, the macular thickness can be represented as a falsely colored topographic map with green and yellow representing normal and near-normal values and areas with progressively increasing retinal thickness being represented by orange, red, and white in agreement with the color-coded scale [4]. DME and its response to treatment are commonly monitored using automated OCT retinal thickness mapping [5]. Standard OCT, however, only offers structural information, and as a result, does not distinguish blood flow within the retinal vasculature and merely offers spatial features. Zones of leakage can be linked to structural changes in the retina by fusing the physiological data from FA with the structural data from OCT, allowing for a more accurate assessment and monitoring of the response of DME to various treatment regimens. The decision-making process during the follow-up of patients with DME may be hindered by the occasional unavailability of either imaging modality [6].

With the advent of deep convolutional neural networks (DCNN), which are gradually replacing other approaches in most machine learning tasks like pattern recognition and image recognition, the problem of medical data generation, particularly images, has been of great interest and has thus been extensively studied in recent years [7]. Generative adversarial networks (GANs) are neural network models that simultaneously train their generator and discriminator networks. A network's integrated performance efficiently creates new, realistic image samples [8].

A broad solution to supervised image-to-image translation problems is provided by the pix2pix GAN framework. Its generator takes an image from the input domain and translates it to the target domain by reducing the adversarial loss sent back by the discriminator and the pixel-reconstruction error. Additionally, the discriminator must distinguish between the generator's fake output and the desired ground truth output image until it achieves equilibrium with the generator [9].

We evaluated the effectiveness of a GAN implementing pix2pix image translation from the original OCT color-coded macular thickness map to synthetic FA image translation and the reversal(from original FA image to synthetic OCT color coded macular thickness map translation). Both subjective and objective evaluations of the synthesized images' quality were conducted for each class.

## Materials and methods

### Study population

This study followed the tenets of the Declaration of Helsinki in compliance with applicable national and local ethics requirements. The institutional review board of Faculty of Medicine, Assiut University waived the need for patients' informed consent of this retrospective study. We retrospectively analyzed charts and results of imaging studies for patients from the retina clinic at Assiut University Hospital who had simultaneously undergone same-day FA and OCT with a diagnosis of confirmed or suspected DME, over 31 months (August 2018 to February 2021). Patient demographics and clinical data were obtained from electronic medical records. Previous retinopathy or maculopathy treatment was recorded, and only one examination per patient was analyzed. The exclusion criteria were the following: significant media opacity that obscured the view of the fundus; OCT images with high signal-to-noise ratio, expressed by the device as "TopQ image quality" < 60; and/or vitreoretinal interface disease distorting the OCT thickness map. Patients with concurrent ocular conditions interfering with blood flow, uveitic diseases, or high myopia of more than − 8.0 diopters were also excluded.

### Image acquisition

Anonymized charts of patients with diabetes that contained color fundus photographs and standard 30-degree FA obtained using a conventional fundus camera (TRC-NW8F retinal camera; Topcon Corporation, Tokyo, Japan) were used for diabetic retinopathy grading by two independent retinal specialists (EW and SK). Grading of the retinopathy was based on the Early Treatment Diabetic Retinopathy Study (ETDRS) classification [10]. After classification, the inter-rater agreement between the two graders was calculated. The labeled images were then reviewed by a third party (HA), who identified conflicting images and adjudicated their grades by consensus.

Swept-source OCT was performed using the Topcon DRI OCT Triton device (ver.10.13; Topcon Corporation, Tokyo, Japan) to obtain a 3D macular report view. The report shows a 7.0 × 7.0 mm horizontal box scan of the macular area centered at the fovea. The retinal thickness map is transparently overlaid on a red-free fundus image as a color-coded square that represents retinal thickness measured from the internal limiting membrane to the photoreceptor outer segment/retinal pigment epithelium junction. Normal retinal thickness values are represented in green, while progressively thicker than average values (compared to the normative database) are shown in yellow, orange, red, and white. Meanwhile, thinner than usual values are depicted in blue and violet. A color bar is attached to the map showing the values of retinal thickness for each color ranging from 0 to 500 µm. The relative position of the foveal center in each map is automatically identified by the machine software; sometimes, the certified operator may change the fovea location to correct centration errors.

### Image dataset preprocessing pipeline

All image preprocessing was performed using the Python imaging library (PIL) and Open CV, which is a library of Python bindings designed to solve computer vision problems. Pix2pix uniquely requires paired images across the two domains, which are spatially registered identically to each other. The images must be combined side by side to produce a composite image of width = 2 × height.

Our dataset comprises OCT color-coded macular thickness maps and their corresponding FA frames that feature the same macular area. The image translation problem involves converting the OCT macular thickness map photo to an FA frame, or the reverse (FA frame to macular thickness map image). The prepared images have a digit filename and are in the JPEG format. Each image is 1024 pixels wide and 512 pixels tall and contains both the FA image on the left and the corresponding OCT color-coded macular thickness map image on the right. We used the fovea location as the reference landmark to crop identical macular areas in both image domains. To locate the fovea in FA frames, we implemented foveal avascular zone (FAZ) segmentation using a contour detection technique, and then used the FAZ centroid as the presumed fovea location.

### Preparation of OCT images

All available anonymized 3D macular reports were digitally named and cropped to the color-coded macular thickness map. Then, all left eye maps were mirror-imaged to look as right eye maps. This horizontal flipping of the images serves as an image augmentation technique so that all images appear as fundus images of the right eye. Automated cropping of the resulting images to the color-coded macular thickness square was implemented using a Python script by iterating over pixel values and locating the outlines of the colored square overlaying the red-free (gray-scale) fundus photograph. The algorithm scanned a central squared area to avoid errors introduced by scanning the color bar and reducing the computational cost. The colored square center is considered to be the presumed foveal location. The colored square is finally resized to 512 × 512 pixels JPEG image format and saved.

### Preparation of FA images

Mid-phase frames (2–4 min) were considered for the FA images. FA frames in this phase depict retinal perfusion details and areas of retinal edema before masking by excessive fluorescein leakage can occur. The selected frame of each eye is a digit named after the corresponding OCT map name and resized to correct for the difference in magnification between the fundus camera and OCT machine images. All left eye frames were then mirror-imaged to look like the right eye frames. To automatically segment the FAZ, the FA frames were converted into grayscale, and then binary thresholding was applied over a selected squared area slightly shifted to the left half of the image. This was done to minimize possible segmentation errors by dark frame outlines and reduce unnecessary computational costs. The segmentation algorithm converts the image to black and white while adjusting the threshold to highlight the FAZ and facilitate contour detection. Thresholding turns the border of the FAZ, with all contained pixels having the same intensity into a white area. The algorithm can detect the borders of the FAZ from these white pixels. The black pixels, having a value of 0, were perceived as background pixels and ignored. The coordinates of the FAZ contour points are detected by an OpenCV function (findContours() function). This allowed the localization of the centroid of the FAZ, which was presumed to be the foveal location. As FA images were previously corrected for magnification difference, the centroid of the FAZ was then used as the reference point for cropping the original FA frame to a square equivalent in dimensions to the final cropped OCT macular thickness colored square and saved as 512 × 512 pixels JPEG image. We also prepared a separate reference folder (named "reader's folder") for each eye FA frame containing four images: the first shows the colored fundus photograph, the second shows the FA frame used, the third is the same FA frame with the position of the foveal mark, and the fourth is the binary threshold abstract image showing the segmented FAZ as a white area over a black background with the centroid mark used as the reference point for the presumed foveal location.

### Preparation of paired training and test dataset

Each OCT image is paired with its respective FA and concatenated, giving a 1024 × 512 twin image that represents the same macular area centered on the fovea. This image registration is mandatory during the implementation of the pix2pix GAN. The concatenated image of each eye was added to the corresponding reader's folder. Images in each reader's folder were used to revise the quality of the automated fovea localization algorithm by one of two experienced OCT image readers (MS and WS).The readers reviewed the images in each reader's folder and were asked to subjectively evaluate the overall quality of the centration of cropped images on a scale of 1–5 (1 = excellent, 2 = good, 3 = normal, 4 = poor, and 5 = very poor). The readers were also asked to isolate image pairs with poor or very poor cropping quality (non-identical macular areas or eccentric fovea location). These images were cropped manually, concatenated, and the resulting pairs were reviewed by a third party (HA) until the desired quality of image pairs was reached using all available images.

**Fig. 1:**
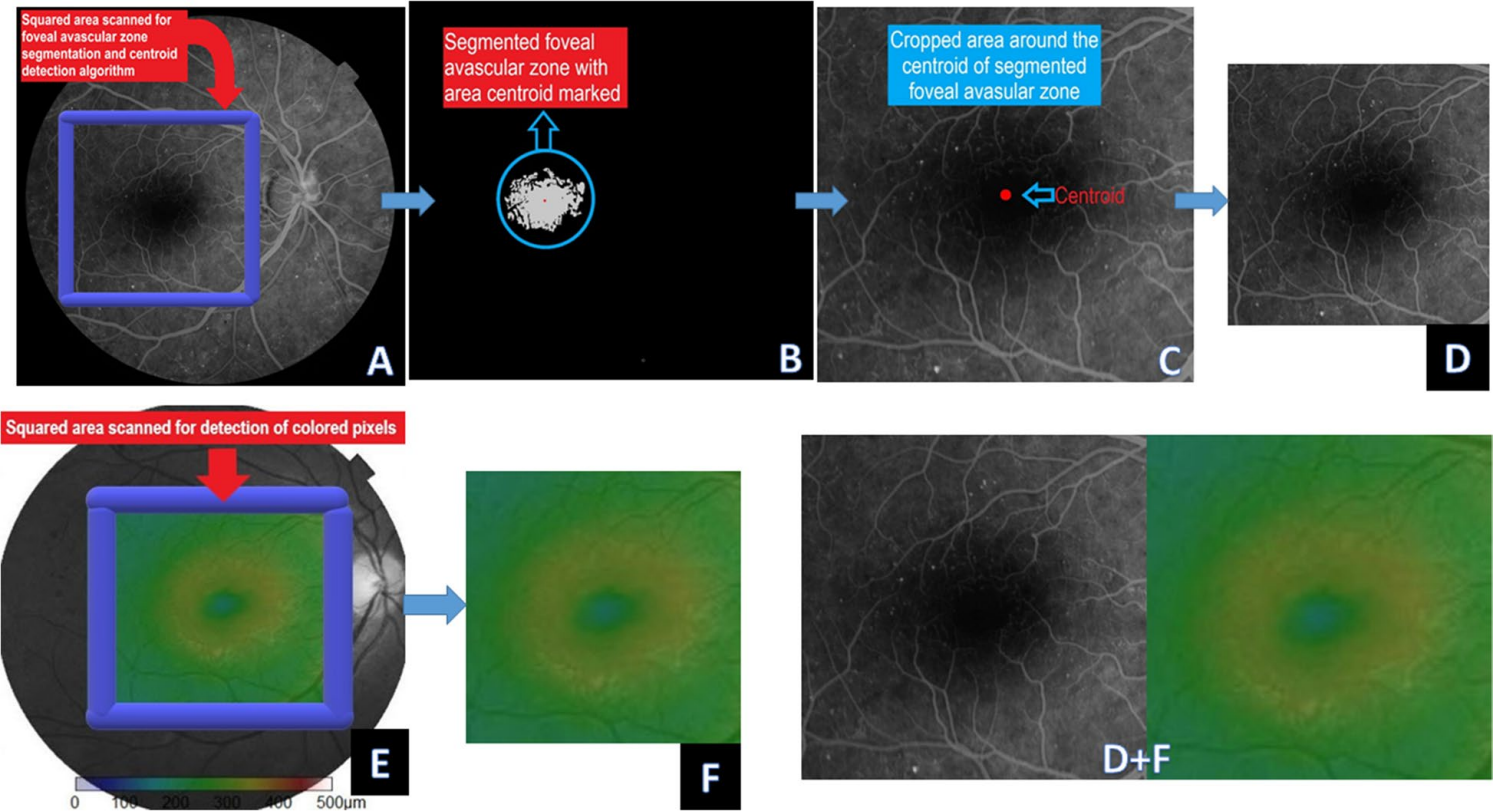
Image preprocessing pipeline: **A** Area scanned for detection of the foveal avascular zone outline in fluorescein angiography frames. **B** Binary image of the foveal avascular zone (white) with its centroid marked (red dot). **C** Original fluorescein angiography frame cropping centered at the centroid location. **D** The cropped, resized fluorescein angiography frame. **E** Area scanned for detection of colored pixels of optical coherence tomography colored-coded macular thickness map. **F** Cropped macular thickness map square. **D** + **F** The final concatenated image

The preprocessing step yielded 1195 paired images as the original dataset. At this point, we manually selected 110 paired images that equally represented all DR stages available in the original dataset to be used as a test set, and the remaining images were used for training the pix2pix network(1085 images). Each image in the test set was saved in a digit-named folder that contains a copy of the original unprocessed image. Figure 1 shows the preprocessing steps for preparing the dataset used in this study.

### Image synthesis

#### Pix2pix GAN architecture

Pix2pix is a conditional GAN in which a target image is generated conditional on a given input image. In this case, the pix2pix GAN changes the loss function such that the generated image is plausible in the content of the target domain and is a plausible translation of the input image. The network is made up of two main pieces known as the generator and discriminator. The generator transforms the input image to obtain an output image. The discriminator measures the similarity of the input image to an unknown image (either a target image from the dataset or an output image from the generator) and tries to guess if it is produced by the generator. The generator is updated to minimize the loss predicted by the discriminator for the generated images [11]. The adversarial loss influences whether the generator model can output sharp images that are plausible in the target domain; using this loss function alone introduces visual artifacts, whereas the use of the mean absolute pixel difference loss between the generated translation of the source image and the expected target image (L1 loss) alone processes blurry images. As such, the combination of L1 loss and adversarial loss is controlled by a new hyperparameter lambda (generator loss = adversarial loss + lambda × L1 loss), which is set to 100, giving 100 times the importance of the L1 loss than the adversarial loss to the generator during training. This combination of loss functions can effectively reduce these artifacts.

More details about the model architecture are mentioned in our previous published work [12].

We implemented the same pix2pix model architecture originally proposed by Isola et al. [9] with minor modifications needed to generate color images of 512 × 512 pixels.

**Fig. 2:**
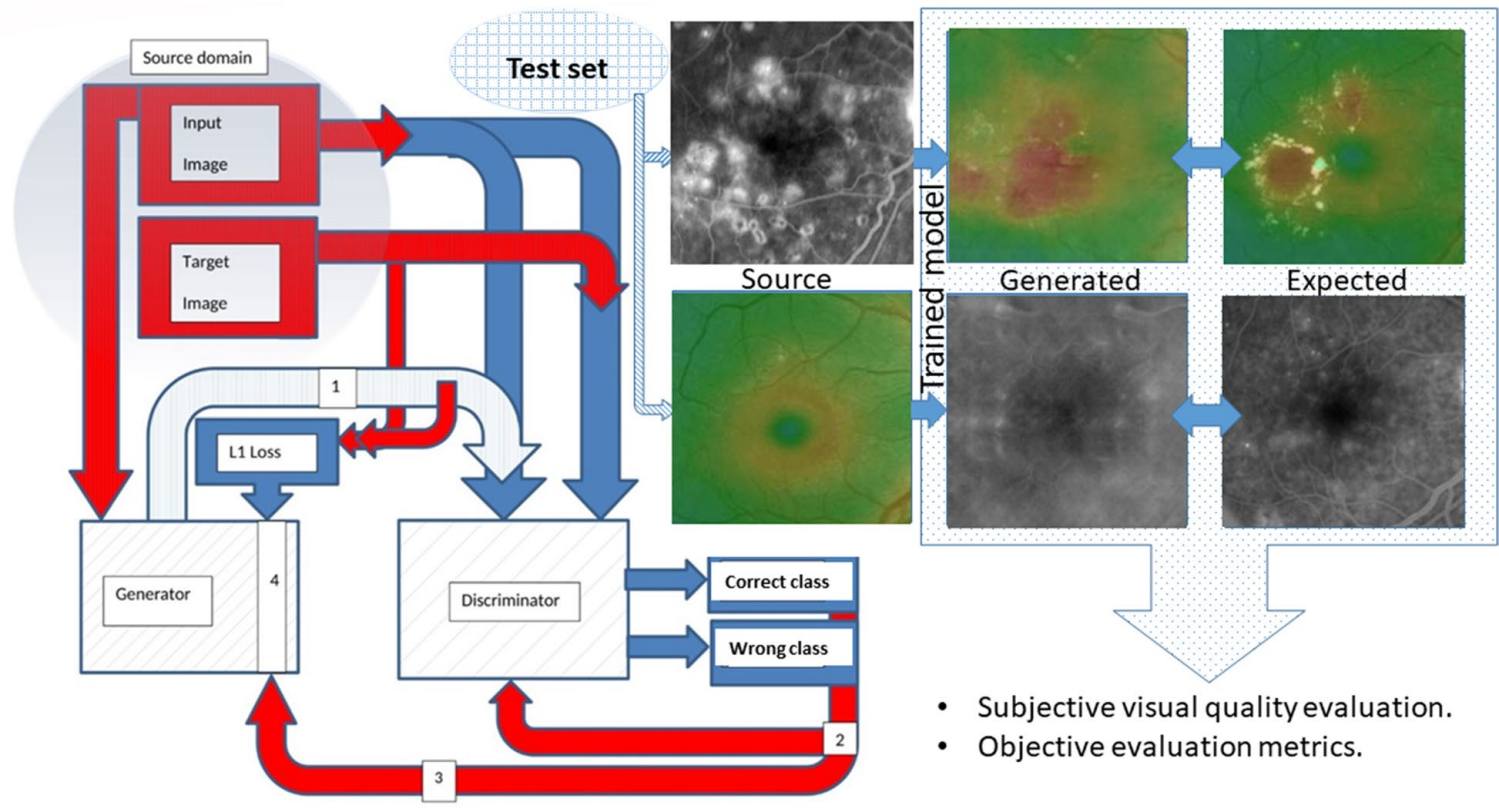
A simplified plot of the proposed composite pix2pix model outlining its main components and workflow (created by Hazem Abdelmotaal). 1: Generated image. 2: Discriminator (L1) loss. 3: Adversarial loss. 4: Composite loss function (generator loss = adversarial loss + lambda (100) × L1 loss). L1 Loss = mean absolute error between the generated image and the target image

We used Keras 2.3.1 a and Tensorflow 2.0.0. [13]; the proposed model architecture is illustrated in Fig. 2.

#### Pix2pix GAN training

Two identical pix2pix GAN models were trained using all available images of the training set (the test set images were set aside from the original dataset as described). One model was trained for FA to OCT macular thickness map translation, and the other was trained in the reverse direction to synthesize FA images from the corresponding OCT macular thickness maps. The model with its weights were saved regularly each 10 training iterations to generate sample images for quality assessment. Consequently, we obtained 20 saved generator model files with their weights for each model task.

#### Selection of synthesized images with the best quality

Each of the 20 saved generator models was loaded and used to make an ad-hoc translation of source images in the training dataset for subjective or objective assessment [12]. To select the generator epoch that produced the best image quality, we used the Fréchet inception distance (FID) score, [14] which is a metric that calculates the distance between feature vectors calculated for original and generated images. A lower score indicates better performance. The saved trained generators were used to synthesize image samples in each direction (from OCT to FA and the reverse) using all test set images after cropping each image pair to obtain each image class separately. The FID score was calculated for images obtained by each of the 20 saved generators per image class. The best performing generator was used for image synthesis, and the synthesized image quality was evaluated subjectively and objectively.

### Subjective visual quality analysis

All test set images were used to obtain synthesized images using the selected trained model epoch in each direction of image translation (from true FA to synthesized OCT macular thickness map, and from true OCT macular thickness map to synthesized FA frame) using the relevant model trained for each translation task. The resulting synthesized image was saved with its corresponding ground-truth counterpart in a separate folder. Each of these images was visually evaluated for global consistency and content by the same experienced observers (SK and EW). They subjectively evaluated the overall quality of the synthesized images on a scale of 1–5 (1 = excellent, 2 = good, 3 = average, 4 = poor, and 5 = very poor). The quality of the original images was used as the standard for a score of 1. They were also asked to report the overall features and clinical usefulness of each class of synthesized images compared to the corresponding ground truth images. We also independently supplied two other image readers (WS and MS) with a patch of 11 ground-truth test set OCT images(10% of the test set volume) and the corresponding shuffled synthesized FA frames and asked them to combine each image with its probable synthesized counterpart. The readers' accuracy and the time needed to submit their answers were recorded. This was repeated using the remaining test set volume and the corresponding synthesized images in 10 image patches until all the test images were provided to the readers. The process was repeated after shifting to the ground-truth test set FA frames and the corresponding synthesized OCT images.

**Fig. 3:**
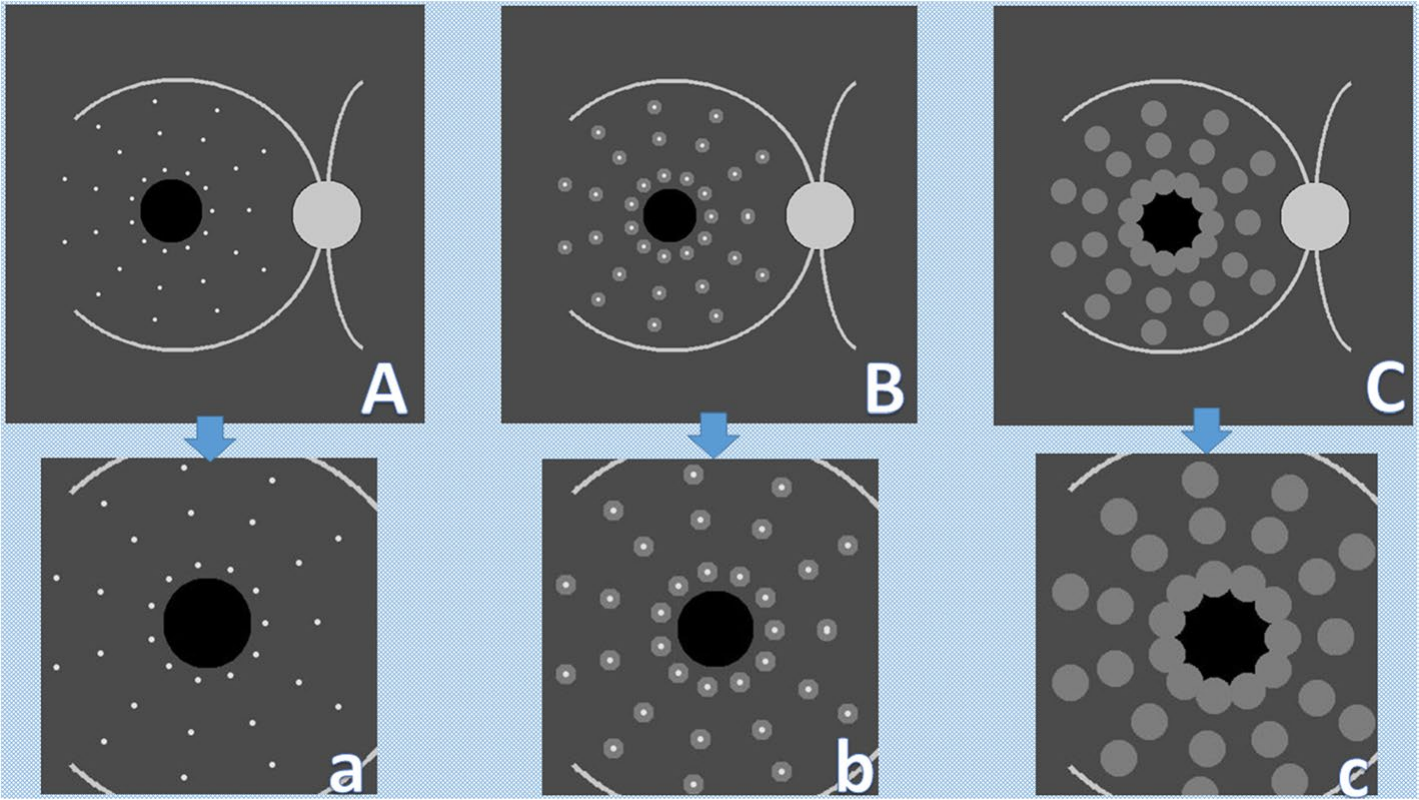
The proposed virtual fluorescein angiography frames for testing the optical coherence tomography macular thickness map generator. (created by Hazem Abdelmotaal). **A** Dotted hyperfluorescence simulating the appearance of microaneurysms without leakage. **a** Image (**A**) after preprocessing similar to the original dataset. **B** Dotted hyperfluorescence simulating the appearance of microaneurysms with surrounding focal leakage. **b** Image (**B**) after preprocessing similar to the original dataset. **C** Dotted hyperfluorescence simulating the appearance of microaneurysms with surrounding diffuse leakage. **c** Image (**C**) after preprocessing similar to the original dataset

Hand Crafted features (HCFs) refer to properties derived using various algorithms using the information present in the image itself such as edges and corners [15]. HCFs were commonly used with "traditional" machine learning approaches for object recognition and computer vision like Support Vector Machines. Convolutional neural networks (CNNs) typically do not have to be supplied with such handcrafted features, as they can "learn" the features from the image data. Instead, we suggested the use of HFCs as a testing tool for obtaining deeper subjective insight into the model performance during FA to OCT macular thickness map image translation. We supplied the chosen trained model with three macular diagrams prepared by PIL that represent abstract diagrammatic drawings of the right macula with dots representing hyperfluorescent microaneurysms with increasing degrees of surrounding hyperfluorescence mimicking leakage. The diagram was designed so that the presumed microaneurysms were distributed in equidistant concentric zones around the presumed FAZ to help understand the model behavior. These virtual FA maps were subjected to cropping in the same steps in the preprocessing before feeding them to the FA to OCT generator. The synthesized virtual thickness maps are presented in this paper. The virtual FA images are shown in Fig. 3.

### Objective evaluation metrics for synthesized images

To objectively assess the synthesized image quality, all synthesized images of each class using the test set were quantitatively evaluated using the following metrics:

#### *Peak signal-to-noise ratio (PSNR) *[16]

The PSNR measures image distortion and noise level between images; a higher PSNR value indicates higher image quality. PSNR calculates the PSNR ratio in decibels from the two images. This ratio is used to measure the quality of the original image and the resultant image. To calculate the PSNR, the mean squared error (MSE) was used to compare the “true” pixel values of the original image with respect to the synthesized image. The MSE represents the average of the squares of the "errors" between the actual image and the noisy image.

#### *Structural similarity index (SSIM) *[17]

The SSIM index is a perceptual metric that quantifies image quality degradation by measuring the structural information similarity between images, where 0 indicates no similarity and 1 indicates complete similarity. The SSIM extracts three key features from an image, luminance, contrast, and structure; then, the comparison between the two images is performed based on these three features. This metric differs from PSNR, which estimates the absolute errors.

#### *Hamming distance (HD) *[18]

HD can be used to determine the similarity of two images. The HD between two strings of equal length is typically defined as the number of positions at which the corresponding symbols are different. The value of the string (hash value) is derived from each image by image hashing based on the visual contents of an image. This involves examining the contents of an image and constructing a hash value that uniquely identifies an input image based on its visual content. This simple cryptographic image hashing cannot be used directly to compare the contents of two images, because if the images are slightly different, even a couple of pixels will result in a completely different hash. For most human eyes, images that differ by a couple of pixels are essentially the same. To be able to compare images in a way that is more similar to human eyes, we used perceptual hashing algorithms so that the more similar the images, the more similar their hashes are. The perceptual hashing algorithms used in our work involve scaling the original image to an 8 × 8 grayscale image, and then performing calculations on each of the 64 pixels. The result is a fingerprint of the image that can be compared to other fingerprints. We used the image-hash library in Python to compute the hash of an image, and then compared it to find the HD. A value of 0 indicates a similar result. A value between 1 and 10 is potentially a variation. A value greater than 10 is likely to be a different image.

#### *Learned perceptual image patch similarity metric (LPIPSM) *[19]

As visual patterns of images are high-dimensional and highly correlated, the notion of visual similarity is often subjective. Aiming to mimic human visual perception, the most widely used perceptual metrics today, such as PSNR and SSIM, are classic per-pixel shallow measures that assume pixel-wise independence and fail to account for many nuances of human perception. In addition, HD is best suited for comparing binary patterns. LPIPSM is a novel metric used to compute the distance between two images in the CNN feature space using the network's internal activation after being trained for image classification tasks. This amounts to the use of pre-trained weights when training the network, to aim for perceptuality, and is demonstrated to exhibit a high correlation with human perceptual similarity. A higher LPIPSM value means more different images, and a lower value indicates more similarity. We used the LPIPS metric to evaluate the mean distance between pairs of ground-truth images in the test set and their corresponding synthesized images and to plot the distance between some image samples from both domains. We used the trained AlexNet net to compute the distance, as recommended in the original paper, as it provides the fastest and best performance. For the LPIPSM estimation, we used PyTorch 1.0 + and torchvision libraries [20].

### Statistical analysis, computer hardware, and software

All statistical analyses were performed using Scipy (Scientific Computing tools for Python) and scikit-learn (version 0.21.3) [21]. Scikit-learn is a Python module for machine learning built on top of Scipy. Patient data are presented as the mean and standard deviation. A t-test was used to compare the means of the image group metrics. The Mann–Whitney U-test was used for the analysis of the means of the five-point assessment score given by the two readers, and a value of *p* ≤ 0.05 was considered significant. The ability of readers to link true with synthesized images was estimated by accuracy (number of correct decisions made divided by the total number of test examples). Inter-rater agreement was estimated with Cohen’s к. Deep-learning computations were performed on a graphics processing unit (GPU) composed of a personal computer with a GeForce RTX 2060 SUPER graphics card powered by an Nvidia Turing architecture with a CUDA 11.0.126 drive.

## Results

A total of 1195 simultaneous, good-quality FA/ OCT examinations (487 bilateral) met the inclusion criteria. Only one examination was performed in the same patient during the study period. Table 1 presents the characteristics of the study population, DR grading, and treatment details. Inter-rater agreement for DR grading was 0.90 (Cohen к 0.84). This trivial ground-truth grade conflict ensured the presence of robust characteristics that the pix2pix GAN can use for the image features-specific style transfer in the synthesized images.

**Table 1:** Demographics of the study population

Clinical characteristics	Population
Subjects/eyes (n)	708/1195
Mean age (y) ± SD	60.3 ± 8.22
Gender (male/ female ratio)	14:11
Duration of diabetes mellitus (y) ± SD	17.3 ± 6.2
Side (right eye/ left eye)	24 / 22
No retinopathy n (%)	143 (12)
ETDRS severity: n (%)	
• Mild NPDR	383 (32)
• Moderate NPDR	191 (16)
• Severe NPDR	251 (21)
• PDR	227 (19)
OCT morphology: n (%)	
• No edema	215 (18)
• DRT	550 (46)
• CME	335 (28)
• SRD	95 (8)
Maculopathy treatments: n (%)	
• No treatments	263 (22)
• Intravitreal anti-VEGF only	526 (44)
• Intravitreal anti-VEGF and steroids	60 (5)
• Macular laser alone	191 (16)
• Macular laser and intravitreal drug(s)	155 (13)
PDR treatments: n (%)	
• PRP only	132 (58)
• Intravitreal anti-VEGF only	31 (14)
• PRP and intravitreal anti-VEGF	41 (18)
• PPV	23 (10)

*anti-VEGF* Anti-vascular endothelial growth factor, *CME* Cystoid macular edema, *DME* Diabetic macular edema, *DRT* Diffuse retinal thickening, *ETDRS* Early treatment diabetic retinopathy study, *NPDR* Non-proliferative diabetic retinopathy, *OCT* Optical coherence tomography, *PPV* Pars plana vitrectomy, *PRP* Pan-retinal photocoagulation, *SRD* Subretinal detachment

### Dataset preprocessing

After preprocessing, we obtained 1195 paired images as the original dataset. The training set comprised 1085 paired images, and the test set comprised 110 paired images that equally represented all DR stages available in the original dataset. The process of automated cropping of identical macular areas in FA and OCT color-coded macular thickness maps based on the location of the FAZ centroid was not successful, as expressed by the five-point assessment score reported by the two readers. The mean of the scores given by the first reader was 3.38 ± 0.72, and by the second reader was 3.66 ± 0.68. Manual cropping was needed to correct 178 paired images (14.9% of the original images).

### Pix2pix GAN training and generator selection

**Fig. 4:**
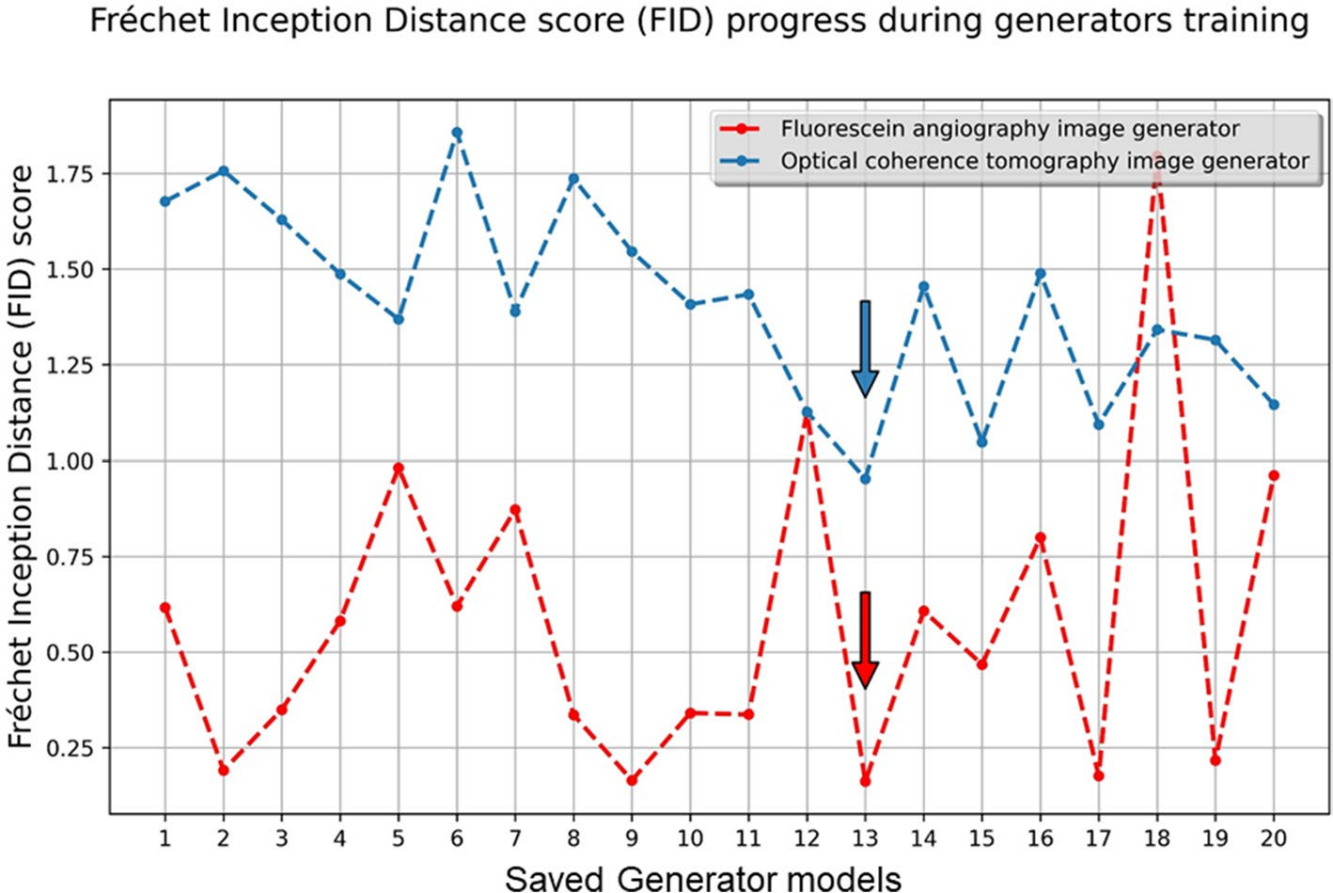
The Fréchet inception distance (FID) score changes during training. The arrows point to the generator models with the best-generated image FID score per class

Pix2pix GAN training for 200 epochs took an average of 14 h for each image class training direction. FID scores tended to improve as training progressed; however, the best FID score was reached at the 13th saved model for both classes of image generation (Fig. 4). The training of generators beyond this point produced lower FID scores, highlighting the importance of this metric for defining the best generator epoch for image synthesis. Therefore, the 13th generator (saved at the 130th training epoch) were selected for further per-class image synthesis in this study.

### Subjective image analysis

**Fig. 5:**
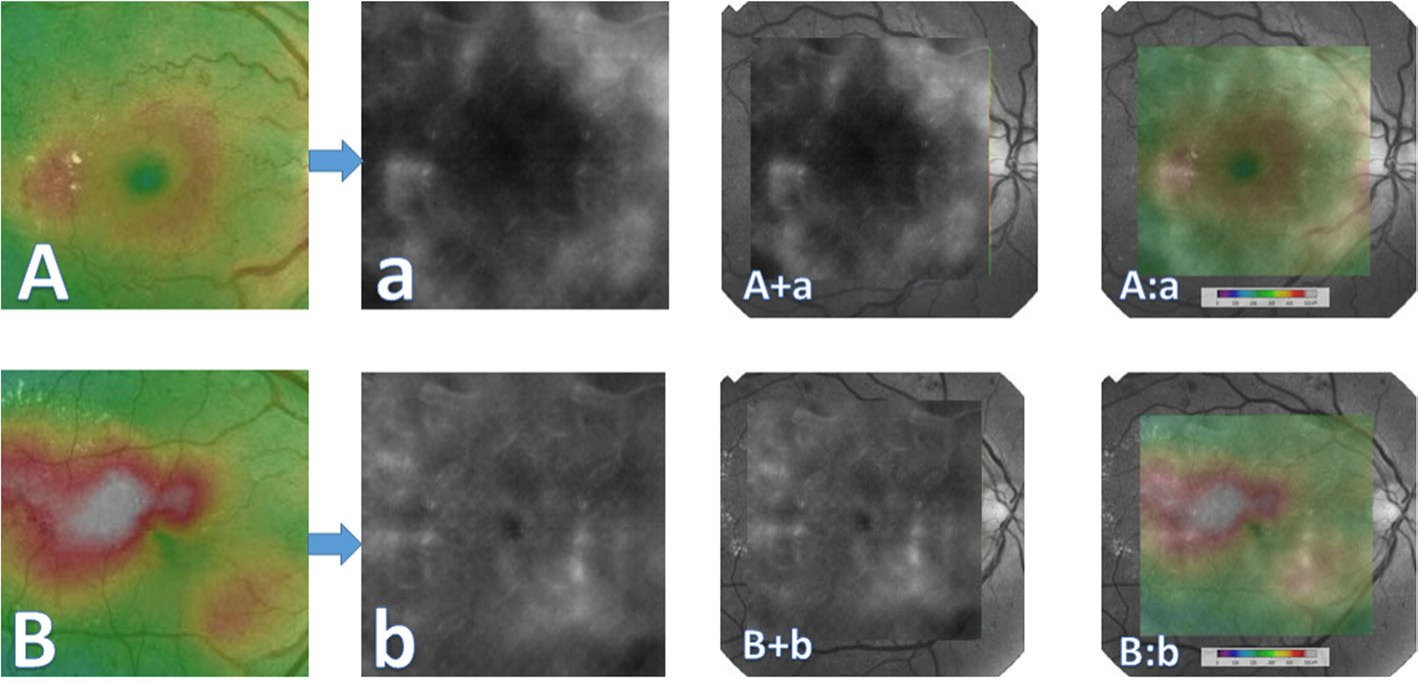
Synthetic fluorescein angiography image examples with various modes of presentation. **A** and **B** Ground-truth cropped optical coherence tomography color-coded macular thickness maps; a and b = generated fluorescein angiography frames; A + a and B + b = generated fluorescein angiography images pasted on their corresponding true red-free optical coherence tomography fundus images; A: a and B:b = generated fluorescein angiography images pasted with transparency on their corresponding true red-free optical coherence tomography fundus images

**Fig. 6:**
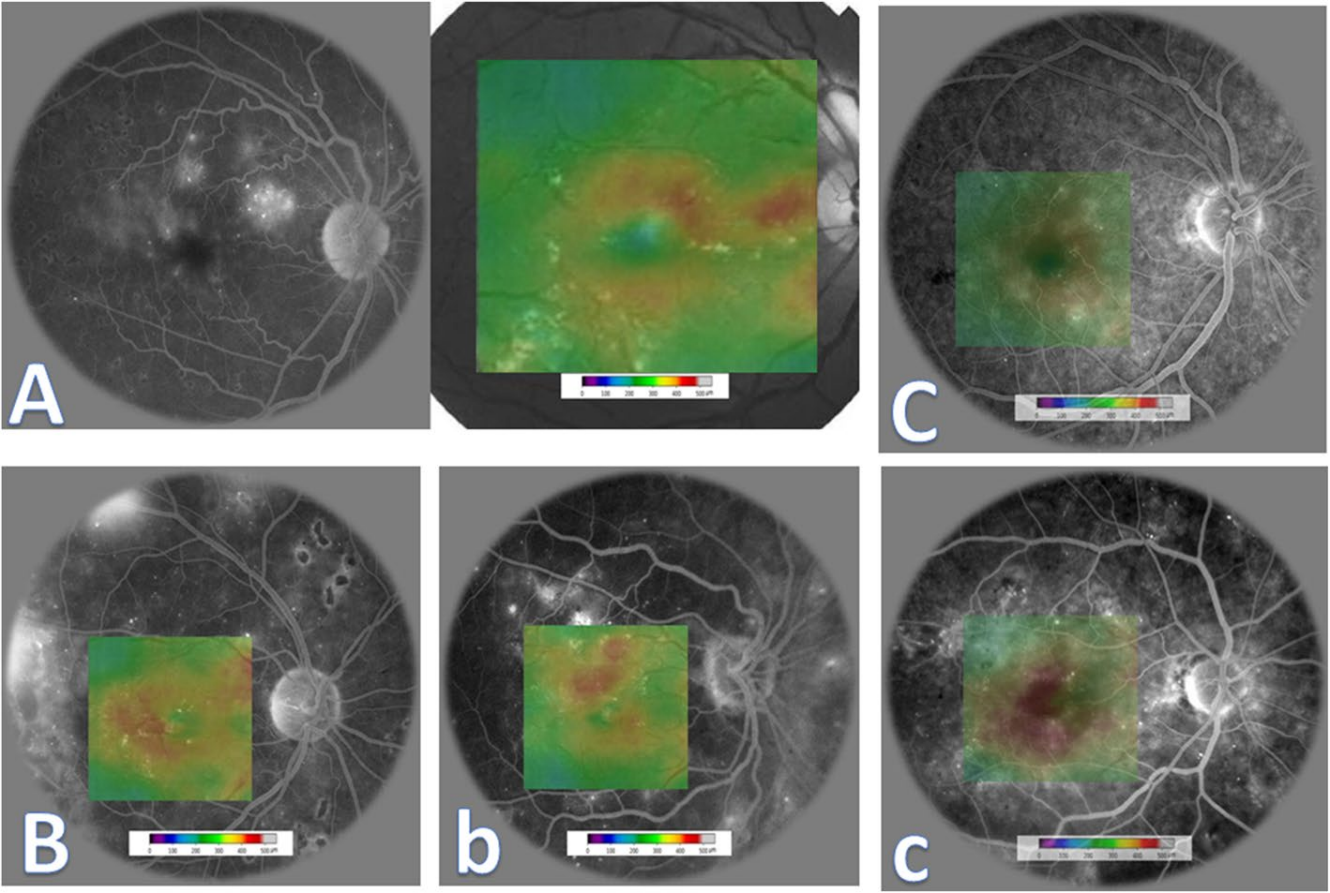
Synthetic optical coherence tomography color-coded macular thickness maps examples with various modes of presentation. **A** Twin image showing the original unprocessed fluorescein angiography frame (left) concatenated with synthetic optical coherence tomography color-coded macular thickness map (right). The synthesized map was pasted on the corresponding optical coherence tomography fundus image provided by a diagrammatic conventional color bar. **B** and **b** Two examples of synthetic optical coherence tomography color-coded macular thickness maps pasted on the corresponding ground-truth fluorescein angiography frames provided by a diagrammatic conventional color bar. **C** and **c** Two other examples of synthetic optical coherence tomography color-coded macular thickness maps pasted with transparency on the corresponding ground-truth fluorescein angiography frames provided with a diagrammatic conventional color bar

Figures 5 and 6 display examples of the synthetically generated images. The images were globally consistent because the model learned to introduce visual content related to linking areas of leakage in FA frames to color codes representing abnormal macular thickening in OCT images. The color code distribution also showed acceptable plausibility. It is also noticeable that the model learned to generate ghost background vessels in the generated OCT maps that were not consistent with the macular vascular tree in the corresponding FA frame. In the other direction, synthesized FA frames lacked background vascular tree and showed no evidence of dotted hyperfluorescence characteristic of microaneurysms but could define areas of focal and diffuse leakage and outline the FAZ.

In the five-point assessment of the overall image quality and clinical usefulness compared to the ground-truth corresponding images, the means of the scores given by the first reader were 2.84 ± 0.31, 2.62 ± 0.96 and by the second reader 2.98 ± 0.55, 2.74 ± 0.29 for the OCT and FA synthesized images respectively. This reflects the good quality of the synthesized images in both classes. There was no significant difference between the classes' average scores given by both readers (*p* = 0.082). This indicates that the models generated plausible expected target image features based on the ground-truth corresponding class features efficiently. Concerning the readers' ability to recombine synthetic and ground-truth images of the other class, they showed robust performance, correctly inferring most of the linking image characteristics in the presented images. When ground-truth FA images were used, the readers achieved correct pairing predictions in 88 images (80%) and 92 images (83.63%) of all 110 challenge images. The average time required by the readers before submitting their answers in the 11-image pairing challenge was 247 ± 45 s and 291 ± 33 s by the first and second reader, respectively. Using the original OCT images, the reader accuracy scores were lower (86 correct pairs [78.18%] and 74 correct pairs [67.27%]), with a statistically significant longer mean time needed to submit the 11 images combining task compared to using the original FA images (395 ± 55 s and 382 ± 16 s, *p* = 0.038 and *p* = 0.014 for the first and the second reader, respectively). The readers reported difficulty in inferring the right pair due to lack of background vascular landmarks in synthetic FA frames and the presence of some areas of unexpected retinal thickening in synthetic OCT images.

**Fig. 7:**
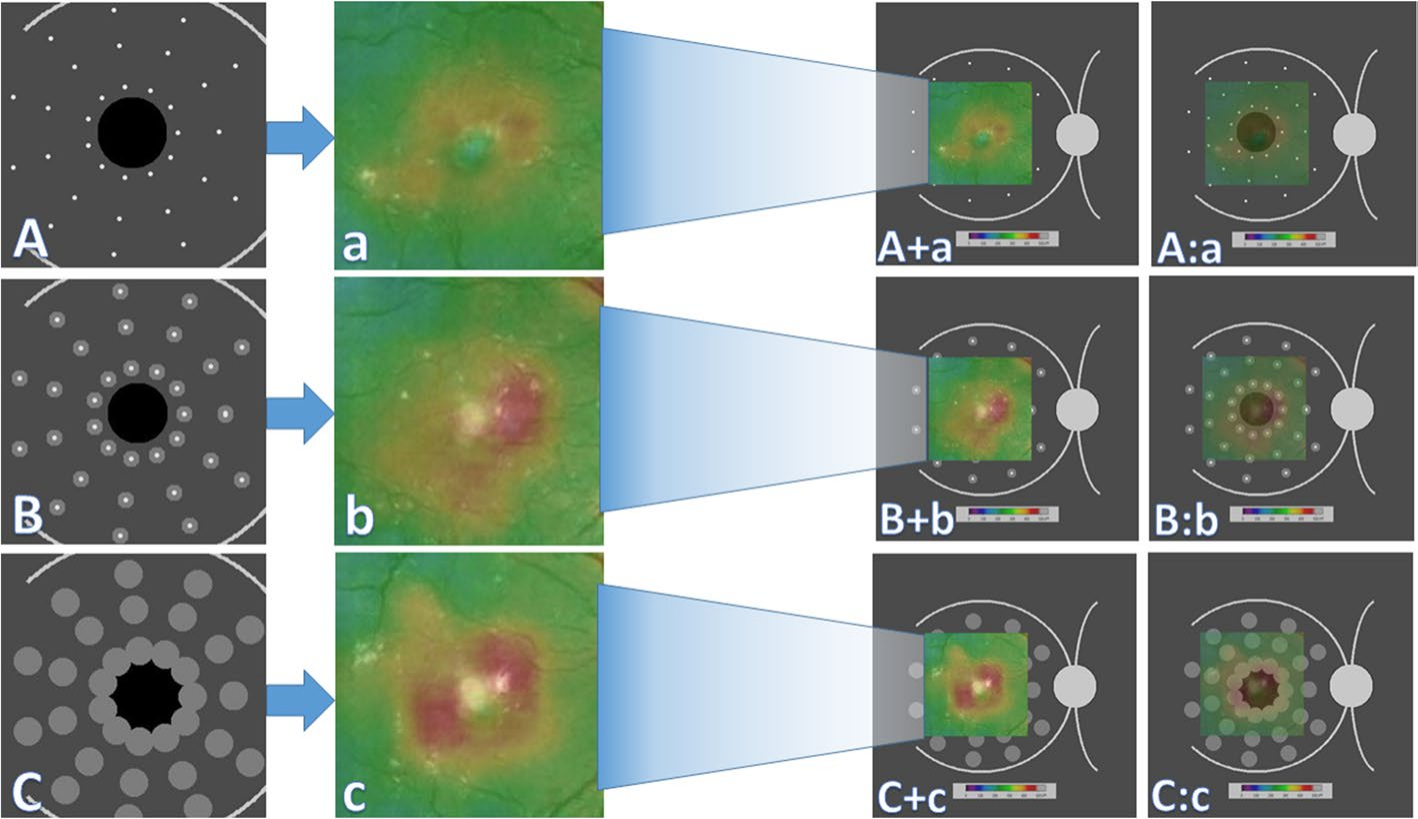
The outcome of feeding virtual fluorescein angiography drawings to optical coherence tomography color-coded macular thickness maps generator model. **A**, **B**, **C** = preprocessed diagrammatic image showing virtual microaneurysms with no or variable leakage; a, b, c = generated thickness maps; A + a, B + b, C + c = synthetic thickness map pasted on unprocessed virtual fluorescein angiography drawing with conventional color bar; A: a, B:b, C:c = generated thickness maps pasted with transparency on unprocessed virtual fluorescein angiography drawing with conventional color bar

When the virtual FA drawings were used to feed the FA to the OCT model, the synthesized OCT maps showed plausible results, as depicted in Fig. 7. The trained model output images showed progressively wider areas of increasing retinal thickness surrounding the presumed leakage. We also noted that the model could map a virtual background vascular tree; additionally, in the mild focal leakage (2nd) frame, the model paid more attention to translating leakage into a greater retinal thickness in the nasal macular region than other macular quadrants despite the symmetric distribution of virtual leaking spots in all quadrants. This may reflect a deep feature in our training dataset or a unique feature that warrants more interest in explaining this attention preference when translating leakage.

**Fig. 8:**
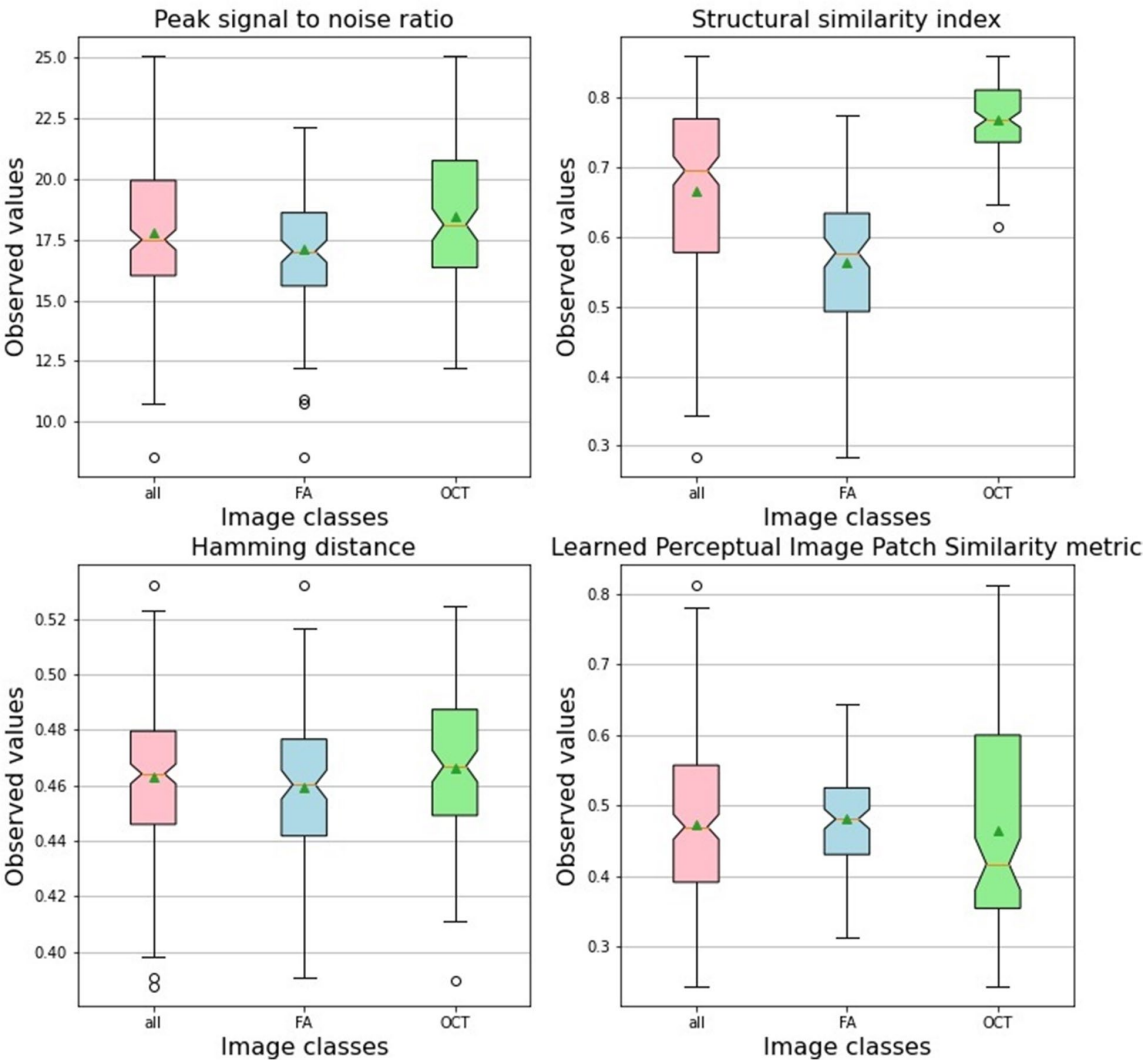
Box-plot of peak signal-to-noise ratio, structural similarity index, Hamming distance and learned perceptual image patch similarity metric between the generated image sample and an equivalent sample of all available test images. Images were synthesized by best generator performance according to the best Férchet Inception Distance score. The notches in the box plot represent the confidence interval around the median. The mean is marked by a triangle. All synthetic images; FA, synthetic fluorescein angiography images; OCT, synthetic optical coherence tomography color-coded macular thickness maps

### Objective image evaluation metrics

**Fig. 9:**
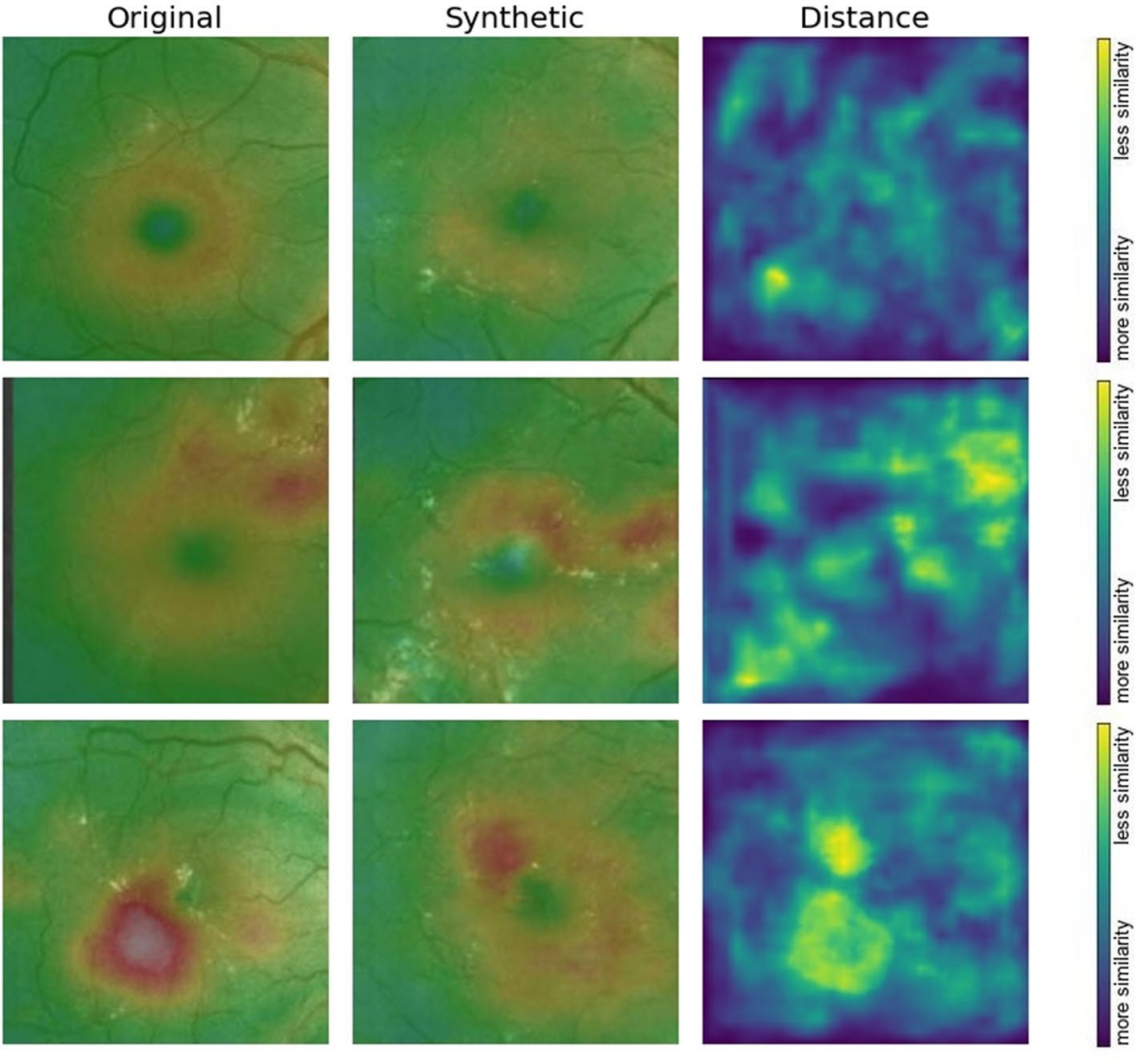
Three examples showing plots of ground-truth and the corresponding synthesized optical coherence tomography color-coded macular thickness maps to show the distance map between some image features as perceived by the Alex Net CNN feature space using the network's internal activation after being trained for image classification tasks. The figures demonstrate the perceptual efficiency of learned perceptual image patch similarity metric in finding image differences

**Fig. 10:**
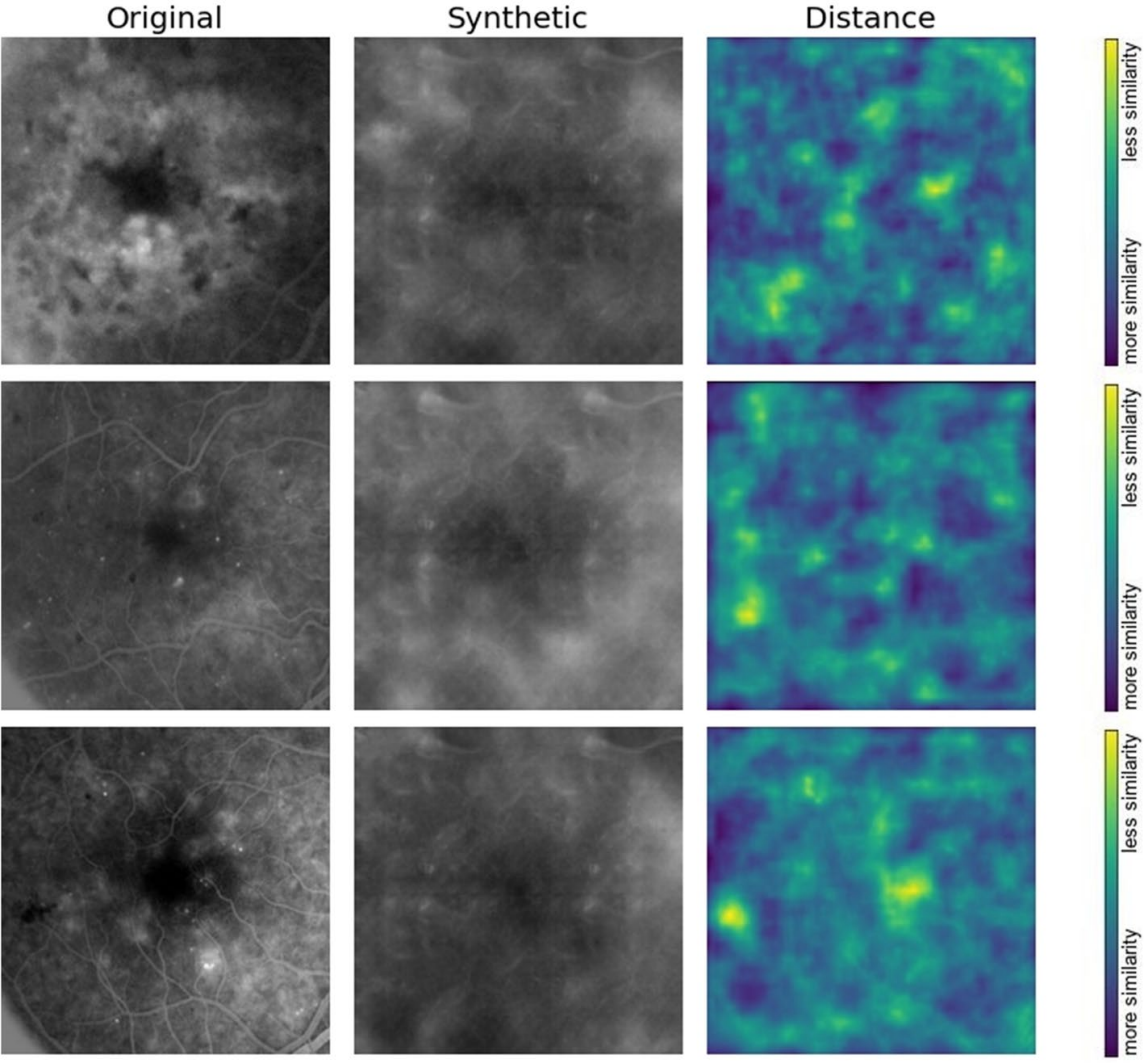
Three examples showing plots of ground-truth and the corresponding synthesized fluorescein angiography images to show the distance map between some image features as perceived by the Alex Net CNN feature space using the network's internal activation after being trained for image classification tasks. The figures demonstrate the perceptual efficiency of learned perceptual image patch similarity metric in finding image differences,

All available ground truth test set image samples in each class were used for pairwise comparison with an equivalent number of synthesized images of the same class by calculating the PSNR, SSIM, HD, and LPIPSM scores. As all the quality scores had a normal distribution according to the Kolmogorov‒Smirnov test, data were expressed as mean ± standard deviation (Fig. 8, Table 2). The generated OCT thickness maps showed a significantly higher (*p* = 0.01) PSNR value, indicating less distortion and lower noise when compared to the synthesized FA images. The SSIM showed that the generated OCT maps also had better structural information similarity with their original counterparts when compared to generated FA images; however, this difference was not statistically significant. The values of HD indicate the presence of variation that was clinically significant in the OCT thickness maps synthesized images (*p* = 0.05). The LPIPS metric showed that the mean distance between pairs of ground-truth images in the test set and their corresponding synthesized images in both image groups was generally below 0.5, with no statistically significant difference, indicating a tendency toward image similarity. In addition, we plotted the ground truth and the corresponding synthesized image samples from each image domain to show the distance map between some image features as perceived by the Alex Net CNN feature space using the network's internal activation after being trained for image classification tasks (Figs. 9 and 10). The figures demonstrate the perceptual efficiency of this metric in determining the image differences. In addition, it confirms that our model generalized properly and did not trivially memorize the training set samples. However, the synthetic images showed areas of unrealistic leakage or retinal thickening compared to the ground-truth images.

**Table 2:** Characteristics of generated image samples compared to their corresponding original images of the same class using the test set

Image class	OCT macular thickness map	Fluorescein angiography	*P-* value
**PSNR**	18.47 ± 2.70	17.10 ± 2.55	**0.01**
**SSIM**	0.77 ± 0.05	0.56 ± 0.11	4.33
**HD**	0.48 ± 0.06	0.43 ± 0.33	**0.05**
**LPIPSM**	0.45 ± 0.14	0.48 ± 0.75	0.25

Data are presented as mean ± standard deviation. Bold entry denotes a significant *P-*value

*HD* Hamming distance, *LPIPSM* Learned perceptual image patch similarity metric, *PSNR* Peak signal-to-noise ratio, *SSIM* structural similarity index

## Discussion

Both FA and OCT are complementary in diagnosing the type and extent of DME [22]. FA has been shown to improve the accuracy of photocoagulation treatment planning for clinically significant macular edema [23]. Also, OCT macular thickness maps have been widely used as a guide for macular laser therapy for DME [24].

The present study showed that applying the pix2pix GAN to either the original FA or original OCT color-coded macular thickness maps can efficiently generate images with plausible quality in the other image domain. To date, no previous study has reported the use of pix2pix GAN in this type of image translation task. The basic pix2pix framework has been used for medical image denoising, reconstruction, segmentation, and amplification of the original dataset [12, 15, 25, 26]. In our study, we developed and evaluated the use of this network to obtain synthetic, clinically useful OCT and FA images that when used in concert, can assist the retinal specialist in decision making while treating patients with DME in case of machine failure/unavailability (either OCT or FA) or situations such as the presence of limited pupillary dilatation or significant media opacity that prevent performance or compromise the quality of FA, while OCT can still be performed with satisfactory image quality. The generated FA images are also useful in patients with multiple comorbidities in whom fluorescein injection may be precluded because of its potentially serious side effects. The potential application of this technique is promising after evaluation of the generated images by retina specialists and many image metrics, including difference detection by trained CNN.

The state-of-the-art in multi-modal retinal images registration is combining feature-based and intensity-based image features [27]. Even the most recently reported hybrid registration framework [28] cannot be used in the preparation of our paired dataset due to the obscuration of the vascular tree by the destructive overlay of color codes in macular thickness maps. We used the FAZ centroid localization algorithm to minimize human effort and errors in the automated preparation of our dataset, permitting preprocessing of any number of image pairs with the least need for manual assistance. The outcome of this preprocessing algorithm was satisfactory after reviewing the image pairs by human readers and allowed automated preprocessing of 84.1% of the original dataset. As different reference standards were used, our preprocessing algorithm cannot be directly compared to other multimodal fundus imaging registration techniques [29].

Fujioka et al. demonstrated that, with increasing epoch number during training of a deep convolutional GAN, the final image quality increased. However, they postulated that overlearning may occur if learning extends beyond the ideal number of learning iterations, decreasing image quality [30]. Our observations corroborate these results. Many studies used subjective scores by experienced image readers to select best image generators [30–32]. However, inter-and intra-individual image reader variations may introduce estimation bias. We used the FID score as an objective metric to assess the generated image quality during training. Monitoring the FID score periodically facilitated accurate selection of the best performing generator epoch.

De Carlo et al. [33] used an overlay technique to simulate vascular leakage in optical coherence tomography angiography (OCTA) in eyes with DME by overlying OCT angiograms with the corresponding OCT thickness maps, using FA as the comparative norm. Their technique showed low sensitivity for the detection of leaking microaneurysms (26.1%). Their overlay technique mandates the availability of the OCTA imaging modality and cannot provide FA frames from the OCT domain. Our method could provide synthetic OCT color-coded macular thickness maps and FA images with plausible subjective and objective qualities in FA and OCT domains, comparable to other studies implementing pix2pix networks for other image datasets [31, 32].

To eliminate possible concerns that the model generated images that were too similar to or simply averaging the original training image features, and to ensure the generalizability of the model, we implemented all subjective and objective analyses on the independent test set [34]. The overall subjective evaluations of the synthesized OCT and FA images were acceptable by both readers. Our results demonstrated that, although retina specialists reported difficulty in identifying original OCT macular thickness maps that correspond to synthetic FA images due to the lack of vascular trees in the synthesized FA images, these images can still convey clinically useful information about possible areas of leakage and outline of FAZ. Dmuchowska et al. [35] stated that it is not possible to detect the FAZ outline or size based solely on the measurement of thickness and retinal structure evaluation using OCT. However, in our generated FA images, the outline/size of the FAZ can be depicted. The validity of these synthetic FAZ plots requires further assessment, which is beyond the scope of our work.

Kozak et al. [36] noticed some discrepancies in the findings between OCT and FA in the detection of microaneurysms. They observed cases in which FA showed obvious patterns of macular leakage but lacked any corresponding changes in retinal thickness on OCT (3.85% of eyes). They also reported the reverse phenomenon in which, in some cases, FA can miss intraretinal fluid, especially subretinal fluid apparent by OCT (1.17% of eyes). Theoretically, this may not interfere with the mutual translation of information between both image modalities because of the low prevalence of both phenomena. On the other hand, they reported a high agreement between the two techniques for cystoid macular edema detection (almost 95%). We also excluded cases with tractional DME in the original cohort, as in this patient category, fluorescein leakage is often minimal or absent, which does not correlate with findings on OCT [37].

Targeting microaneurysms using focal photocoagulation is a strong tool for improving macular edema [38]. Based on synthetic FA findings, microaneurysms could not be defined as dots with hyperfluorescence, instead, the model produced areas of focal and diffuse hyperfluorescence without dotted hyperfluorescence characteristic of microaneurysms. This may be caused by the use of mid-phase FA frames in the preparation of our dataset, in which the dotted hyperfluorescence of microaneurysms is relatively masked by hyperfluorescence caused by progressive leakage. However, the authors suggested that the location of microaneurysms and leaking vascular anomalies could be determined in relation to the path of the macular vessels, guided by using the colored or red-free OCT fundus image with the generated FA frame in concert. The merging of information from both imaging modalities may assist in the localization of leaking vascular abnormalities. In addition, previous reports have shown that some microaneurysms with dye leakage shown by FA did not completely overlap with retinal thickening on OCT [39, 40]. The combination of FA and OCT thickness maps is informative in determining the microaneurysms that are responsible for retinal swelling and treating focal DME.

The presence of artifacts may be partially due to the low amount of data and the transposed convolutions used in the generator architecture that are responsible for concatenating low-level features in the encoding path with symmetrical high-level features in the decoder path [41]. Also, the absence of vascular trees in synthesized FA images and the presence of unrealistic vascular trees in generated OCT images are partly due to the destructive nature of the color-code overlay that obscures the underlying vasculature in the background red-free OCT image domain. Obscuration of vascular markings prevents the model from high-dimensional mapping of macular vessels into the latent space where high-level features are extracted from individual pixels. Consequently, when this latent space is used to morph the original images into new analogous images, the new images appear devoid of blood vessels. When FA frames are used as the source domain, the blood vessels can always be depicted in synthesized OCT images showing evidence that the network always tries to hallucinate missing information in the target domain [42]. This finding can be identified in all model output OCT images, including diagrammatic macular drawings. The model output of diagrammatic macular drawings showed increased retinal thickening corresponding to the expected leaking area surrounding the virtual microaneurysms, with especially remarkable macular thickening in response to perifoveal nasal macular microaneurysms. This response may be related to general findings in training data and/or may reflect a deep feature extracted by the network, thus documenting a clinical finding. We surmised that perifoveal nasal macular microaneurysms are generally more prone to leakage or are more resistant to treatment modalities than aneurysms elsewhere. This finding is consistent with the zone-specific response reported by Vemala et al. [43] who reported that clinically significant macular edema and the inferior perifoveal zone are the most responsive and the parafoveal superior and nasal being the least responsive to macular laser photocoagulation, which was used to treat 29% of our cases.

Yu et al. [32] documented better PSNR and SSIM when using the pix2pix framework than the Cycle-GAN. We also postulate that unpaired training with the Cycle-GAN does not have a data fidelity loss term; therefore, preservation of small abnormal regions during the translation process is not guaranteed.

Because we were interested in generating images with clinically useful quality rather than synthetic image data augmentation, the use of Alex Net to create the LPIPS distance map and metric was more informative to pinch-mark network performance and detect the differences in generated image features compared to ground-truth fellows instead of the traditional use of the classification performance alone into original and synthesized image classes. Additionally, DR staging was used to ensure the diversity of our original dataset and allow equal representation of all DR stages during the manual selection of the test set to ensure generalizability.

Our study had some limitations. Generative models are always limited by the information contained within the training set and how they capture the variability of the underlying real-world data distribution. Generalization of the generator output could be further improved by including more training images. In addition, our method to prepare registered pairs of OCT and FA images was not completely successful, and manual correction of errors was needed. This can be avoided by using the same machine to obtain simultaneous FA and OCT images, which guarantees simple and accurate co-localization of images. Additionally, the dependence of scan placement by the operator at the presumed foveal center during OCT acquisition may cause potential errors in poorly cooperative patients or patients with significant retinal disease. Finally, we did not investigate the impact of changing the value of the hyperparameter lambda, which combines the L1 loss with the adversarial loss. Modulation of this hyperparameter may have limited blurring noticed in synthetic FA images and could also help to translate the image of tiny blood vessels present in the background of the color-coded OCT maps to the synthetic FA domain.

The feasibility of obtaining synthetic FA frames from original OCT macular thickness maps or the reverse may provide a helpful image translation platform in situations in which either imaging modality is inaccessible or inconvenient. Future modifications of our work may theoretically allow this image translation platform to find its place in this niche. These modifications include the construction of more customized models (different generator architectures or the use of an ensemble model that combines the output of multiple generator architectures and investigating different combinations of the discriminator and generator losses). Additionally, we recommend experimenting with the use of early and late FA frames and performing image acquisition simultaneously using the same machine capable of precise co-localization of both imaging modalities.

## Data Availability

All data files used to support the findings of this study are available from the corresponding author upon request.

## Abbreviations

pix2pix GAN: Pix2pix generative adversarial network
OCT: Optical coherence tomography
CNNs: Convolutional neural networks
DCNN: Deep convolutional neural network
DME: Diabetic macular edema
DR: Diabetic retinopathy
ETDRS: Early Treatment Diabetic Retinopathy Study
FA: Fluorescein angiography
FAZ: Foveal avascular zone
FID: Fréchet inception distance
GANs: Generative adversarial networks
GPU: Graphics processing unit
HCFs: Hand Crafted features
HD: Hamming distance (HD)
LPIPSM: Learned perceptual image patch similarity metric
MSE: Mean squared error
PIL: Python imaging library
PSNR: Peak signal-to-noise ratio
SSIM: Structural similarity index
